# Temporal Lobe Cortical Electrical Stimulation during the Encoding and Retrieval Phase Reduces False Memories

**DOI:** 10.1371/journal.pone.0004959

**Published:** 2009-03-25

**Authors:** Paulo S. Boggio, Felipe Fregni, Claudia Valasek, Sophie Ellwood, Richard Chi, Jason Gallate, Alvaro Pascual-Leone, Allan Snyder

**Affiliations:** 1 Berenson-Allen Center for Noninvasive Brain Stimulation, Department of Neurology, Beth Israel Deaconess Medical Center, Harvard Medical School, Boston, Massachusetts, United States of America; 2 Cognitive Neuroscience Laboratory and Developmental Disorders Program, Center for Health and Biological Sciences, Mackenzie Presbyterian University, Sao Paulo, Brazil; 3 Centre for the Mind, University of Sydney, Sydney, Australia; James Cook University, Australia

## Abstract

A recent study found that false memories were reduced by 36% when low frequency repetitive transcranial magnetic stimulation (rTMS) was applied to the left anterior temporal lobe after the encoding (study) phase. Here we were interested in the consequences on a false memory task of brain stimulation throughout the encoding and retrieval task phases. We used transcranial direct current stimulation (tDCS) because it has been shown to be a useful tool to enhance cognition. Specifically, we examined whether tDCS can induce changes in a task assessing false memories. Based on our preliminary results, three conditions of stimulation were chosen: anodal left/cathodal right anterior temporal lobe (ATL) stimulation (“bilateral stimulation”); anodal left ATL stimulation (with a large contralateral cathodal electrode – referred as “unilateral stimulation”) and sham stimulation. Our results showed that false memories were reduced significantly after the two active conditions (unilateral and bilateral stimulation) as compared with sham stimulation. There were no significant changes in veridical memories. Our findings show that false memories are reduced by 73% when anodal tDCS is applied to the anterior temporal lobes throughout the encoding and retrieval stages, suggesting a possible strategy for improving certain aspects of learning.

## Introduction

In a seminal study, Bartlett noted that memories are not literal representations of the past [1]. Instead, “facts” are unconsciously constructed to fit our schemata [2], [3], which can lead to false memories. Whilst constructive memory is an important component of an efficient healthy memory system, and is important for future planning [4], [5], there are obvious benefits if false memories can be reduced temporarily in certain circumstances.

To that end a recent study [6] found that false memories are reduced by temporarily disrupting anterior temporal lobe activity, using low frequency magnetic pulse stimulation (rTMS). This area has been implicated in semantic memory and conceptual labeling [7], [8], [9]. After active stimulation, participants had 36% fewer false memories than they had following sham stimulation, while veridical memory was not affected. This is comparable to the advantage that subjects with autism and semantic dementia – by virtue of a reduction in gist-based memory – show over “normal” individuals [10], [11]. In this study, TMS was applied for 15 minutes after the study phase, that is, after the encoding and before the retrieval test phase.

In this investigation, we are interested to study the influence of transcranial direct current brain stimulation (tDCS) [12] on false memories when the stimulation is applied continuously, before the encoding as well as during the retrieval test phase. We predicted that the reduction in false memories would be larger if local activity modification is done before encoding phase. tDCS is an attractive tool for this goal as it is a non-invasive, safe method to change membrane resting threshold and modify spontaneous activity [12] and therefore modify information processing effectively [13].

A clue for achieving our goal of reducing false memories comes from patients with left anterior temporal lobe dementia who have autistic-like qualities [14], [15], [16]. Individuals with autism and temporal lobe dementia are known for being literal [11], [16], [17], [18], [19] and less susceptible to false memories [10]. On the other hand, the more concept orientated we are, the more we tend to categorize and the more prone we are to false memories [7], [19], [20].

The anterior temporal lobes (ATL), especially the left ATL, are vital for semantic processing, being implicated as the region responsible for conceptual knowledge, labels and categories [16], [21], [22], [23]. When the left ATL is damaged, patients lose their semantic memory and their ability to name or label objects, while retaining the ability to retrieve literal details [16], [23].

For these reasons, we hypothesized that disruption of ATL activity by tDCS would reduce false memories by diminishing our reliance on gist in encoding and retrieval.

## Methods

### Study participants

Participants were recruited by advertising in flyers and notices distributed throughout local universities. We included healthy participants aged between 18 and 30 years. Participants were excluded if they had any neuropsychiatric disorder, current or past history of alcohol or other drug use, were taking any medication acting on the central nervous system or were pregnant. Thirty participants (mean age of 19.8±1.16, 20 females) were enrolled in this study. All subjects were undergraduate students, and naïve to the task. Participants gave written informed consent for the study, and approval was obtained from the local research ethics committee (process approval number 0042.0.272.000-07). The study was carried out to conform to the principles of the Declaration of Helsinki.

### Study protocol

Participants were randomized to receive one of the three types of intervention: 1) anodal (+) left anterior temporal lobe/cathodal (−) right anterior lobe (referred in the text as “bilateral stimulation”); 2) anodal (+) left anterior temporal lobe/cathodal (−) right anterior lobe – however the size of cathodal electrode in this condition was 100 cm^2^, a much larger and more diffuse pad than the standard 35 cm^2^ pad, (referred in the text as “unilateral stimulation”); and 3) sham stimulation. Participants and the evaluating investigators (except the investigators that applied tDCS) were blinded to the treatment condition. All stimulation sessions were carried out by the same researchers and at the same time of the day.

### Cognitive tasks

We used the same task as was used in the recent rTMS study [6]; that is, a modified version of Roediger and McDermott's (1995) paradigm. Participants were instructed to remember three lists of words and told that they would be asked to recognize them later. Each list has a “theme”. For example, one list contains words related to bread (e.g. loaf, sandwich, and so forth), but not the word “bread” per se. In selecting categories of stimuli for the false memory task, two criteria were balanced against each other. We chose categories that had reasonably high false recognition rates and contained words that were closely related enough to allow us to select three critical lures (instead of one) whilst leaving nine study words that would establish the category concept. We included three critical lures per category to maintain sufficient power in the test. Piloting confirmed the efficacy of our test in revealing false memories.

We asked our participants to memorize a series of 27 words, each presented for three seconds on a computer screen. The words were selected from three different semantic categories (e.g. bread, music and doctor). They were then presented with 27 words in succession and were asked to click ‘yes’ if they had seen the word earlier or ‘no’ if not. Specifically, participants were shown nine “true” words (words that they had seen before), nine “false” words (words that they had not seen before) and nine unrelated words (words they had not seen before and were not related to the categories of words). We then analyzed veridical memory for “true” words (words that were presented previously) and false memories (false positives for critical “lure”). This test was performed during and after stimulation with an interval between the recognition phases of approximately 10 minutes.

### Transcranial direct current stimulation

**Figure 1:**
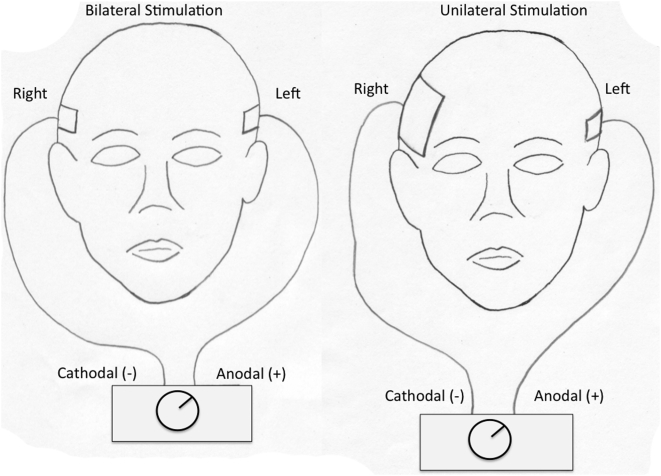
Schematic representation of electrode montages. A represents bilateral stimulation (35 cm^2^ electrodes in the left and right ATL) and B represents unilateral stimulation (35 cm^2^ electrode in the left and 100 cm^2^ electrode in the right ATL).

Direct current was transferred by a saline-soaked pair of surface sponge electrodes and delivered by a specially developed, battery-driven, constant current stimulator with a maximum output of 10 mA. We used electrodes of two different sizes: for the anode (+) electrode, we used a sponge of 35 cm^2^ [12], for the cathode (−) electrode we used the conventional 35 cm^2^ electrode and also a larger electrode of 100 cm^2^ as it has been shown that this large electrode induces a small and nonsignificant effect on cortical activity [24]. The latter electrode configuration was designed to perform a functional monopolar anodal tDCS without relevantly shifting excitability of the contralateral temporal lobe by the cathodal, reference electrode (figure 1).

As aforementioned, participants were randomized to receive three different types of treatment:

1. Anodal stimulation of the left temporal cortex and cathodal stimulation of the right temporal cortex (referred in the text as “bilateral stimulation”). The anode electrode was placed over T3 (using the EEG International 10/20 System) and the cathode electrode over T4. For this condition we used two electrodes of 35 cm^2^.
2. Anodal stimulation of the left anterior temporal lobe and cathodal stimulation of the right anterior lobe – however the cathodal electrode was 100 cm^2^ (referred in the text as “unilateral stimulation”). The anode electrode was placed over T3 (using EEG 10/20 system) and the cathode electrode over T4.
3. Sham stimulation. For sham stimulation, the electrodes were placed in the same positions as in active stimulation; however, the stimulator was turned off after 30 seconds of stimulation. Therefore, the participants felt the initial itching sensation associated with turning on the device, but received no current stimulation for the rest of the treatment period. A recent study showed that this method of sham stimulation reliably convinces the participant they are receiving active stimulation [25 2006].

The rationale for choosing the anterior temporal lobe (ATL) is because this area, especially the left ATL, is vital for semantic processing, being implicated as the region responsible for conceptual knowledge, labels and categories [16], [21], [22], [23]. When the left ATL is damaged, patients lose their semantic memory and their ability to name or label objects, while retaining the ability to retrieve literal details [16], [23].

Regarding the polarity, we decided to use anodal only in the left temporal area. This is because, in our pilot study, using cathodal stimulation over the left and anodal stimulation over the right ATL, we found no differences between active and sham stimulation. Finally, we decided to test two active conditions with reference electrodes of different sizes, in order to test whether stimulation of the contralateral, right, ATL plays a role in modulating false memories.

A constant current intensity of 2 mA (current density of 0.06 mA/cm^2^) intensity was applied for approximately 10 minutes (according to the duration of the task – stimulation was ended when the task was completed). Cognitive tasks were initiated 5 minutes after the start of stimulation as it has been shown that 3 minutes of stimulation is the minimum duration of stimulation in order to induce significant after-effects changes in the cortical excitability [26]. Stimulation with 2 mA (for a single session) has been shown to be safe in healthy volunteers [27].

### Experimental design

After screening and consent, subjects were randomized to one of the three conditions of stimulation (bilateral, unilateral of sham stimulation). tDCS was then started and after 5 minutes of stimulation, the false memory task was initiated (referred in the text as *during stimulation*). Stimulation was then terminated at the end of the false memory task and the subjects were tested again with the same memory paradigm (referred in the text as *after stimulation*).

### Statistical analysis

Analyses were performed using *Stata* statistical software (version 9.2, StataCorp, College Station, Texas). We treated the number of responses as a continuous variable. We therefore analyzed the data using a mixed ANOVA model where the dependent variable was the number of false memories and the independent variables were the condition of stimulation (sham, bilateral and unilateral stimulation) and time course (during or post stimulation). Finally we included the subject ID as a random independent variable to control for the within subject variability. If appropriate, pairwise comparisons were conducted correcting for multiple comparisons using Bonferroni correction. Statistical significance refers to a two-tailed p value <0.05.

## Results

Participants tolerated stimulation well. When analyzing the number of lures as the dependent outcome, the mixed ANOVA revealed no significant interaction effect between condition and time course (F(2,54) = 0.78, p = 0.46). However, there was a significant effect of condition ((F(2,54) = 18.11, p<0.0001), demonstrating that the number of lures was different according to the condition of stimulation. Finally there was no significant effect of time course (F(1,54) = 0.03, p = 0.86); showing that the effects of stimulation were the same during and after stimulation.

**Figure 2:**
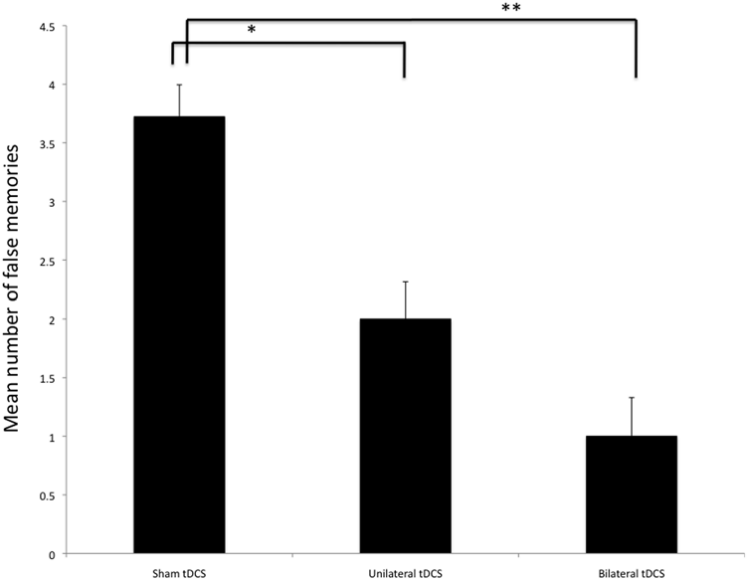
Performance as indexed by number of false memories during sham, unilateral and bilateral stimulation. Columns represent the mean number of false memories and error bars indicate mean standard error.

As the main effect of condition was significant, we then performed pairwise comparisons to compare the different conditions of stimulation (unilateral, bilateral and sham stimulation). Both active conditions were associated with a significant decrease in the number of false memories as compared to sham stimulation (by 73.1%, p<0.0001; by 46.3%, p = 0.0006, bilateral and unilateral stimulation, respectively, corrected p-values). Although, it seems that bilateral stimulation induced larger effects, there was only a trend for a significant difference between these two conditions (p = 0.09) (figure 2).

Finally, in order to assess whether the reduction in false memories was associated with a reduction of veridical memories (suggesting a failure in memory overall), we performed the same model; but at this time using the veridical memories as the dependent variable. There was no significant effect for the interaction term, the main effect of condition or the main effect of time (F<1 for all the comparisons). Table 1 presents mean+SE for veridical memories during and after each type of stimulation). This confirms that the reduction in false memories was not because subjects had an overall poor memory performance during active stimulation.

**Table 1:** Mean number of veridical memories during and after stimulation.

*Veridical Memories*				
tDCS	During		After	
Bilateral	7.6	±0.2	7.8	±0.6
Unilateral	7.7	±0.3	8.3	±0.1
Sham	8.3	±0.2	7.9	±1.0

## Discussion

Our results confirm the notion that modulating activity of the ATL with brain stimulation before or during a given cognitive task is an effective method to change memory processing [6]. In our study, we found evidence that anodal tDCS to the left ATL before the encoding and retrieval phase is effective in reducing false memories while maintaining veridical memory performance unchanged. Our findings support existing evidence that the left ATL is critical for semantic processing [14], [15], [16].

Interestingly, both unilateral and bilateral tDCS (as depicted in Fig. 1) were effective in reducing false memories (see Fig. 2). However, although not significant, the magnitude of effect after bilateral stimulation was larger than that of the unilateral stimulation. To explain this trend, two hypotheses need to be entertained: (1) bilateral tDCS increases the amount of current injected into the left anterior temporal lobe compared with unilateral stimulation because the larger contralateral reference electrode used in unilateral stimulation might increase electrical current shunt; or (2) bilateral tDCS (see Fig. 1) increases the excitatory effects on the left ATL due to the activity of transcallosal fibers.

When compared with a previous 1 Hz rTMS study [6] using the same task and a similar methodology (except for the stimulation timing), our results might appear contradictory. This is because 1 Hz rTMS is associated with a decrease in cortical excitability [28] and anodal tDCS is associated with an increase in cortical excitability [26]. However, we believe that the two results are complementary as the mechanisms of action of these two techniques are quite different. In fact, 1 Hz rTMS induces a large and comparatively focal electrical current that is strong enough to inhibit the main circuits associated with semantic processing, which is hypothesized to increase literal skills [4].

The effects of tDCS are relatively more diffuse than rTMS – anodal tDCS increases activity in a large cortical area, which could unnaturally compete with the semantic centers associated with ATL. This defocusing effect could de-emphasize and reduce the efficiency of the main semantic processing circuits. However, because we did not use brain neuroimaging to study the mechanisms of action of DC stimulation in this study, we can only speculate on the mechanisms underlying the DC effects on false memories. Our hypothesis is based on the notion that individuals start off with more literal perception and memory systems, but that with maturity, conceptual (semantic) processing becomes more highly developed and efficient [17]. Although it seems adaptive to have a high-efficient circuit for semantic processing, it may also lead to false memories when the neural processing in this circuit becomes faster and more efficient than the other, more literal, memory-related neural circuits. In fact, we believe that the undeveloped brain (also perhaps in autism), produces fewer false memories, because the neural circuit related to semantic processing is not as well-developed as in normal adults and therefore the processing is more literal. In this context, anodal tDCS induced an increased activity in a larger network that by competition decreased the natural advantage of a high efficient neural circuit involved with semantic processing, producing a more literal subject such as in autism. Indeed this defocusing effect induced by excitability enhancing anodal tDCS has been shown by other studies [29], [30], [31].

In summary, the interpretation is different for tDCS and rTMS – whereas 1 Hz rTMS can be compared to a virtual lesion experiment, the results with tDCS need to be seen differently. tDCS should be seen as recruiting alternative areas and therefore decreasing inhibition by direct competition with high-efficient cortical circuits associated with semantic processing.

Another potential hypothesis to explain our results is that the decrease in false memories is associated with a decrease in activity in the right temporal lobe [32], [33]. However, was this to be case, we would not have expected unilateral left ATL stimulation to induce any significant changes (unless anodal stimulation of the left hemisphere induced a decrease in right hemisphere activity via transcallosal activity).

Whilst our explanation for the mechanism of tDCS in reducing false memories is tentative, tDCS has shown itself to be a highly effective method, as seen by the performance increase in 73%, as compared to 36% with rTMS. Gist formation [3] interferes with literal retrieval [19]. In our rTMS study, encoding took place prior to stimulation, so participants may have encoded stimuli according to gist. It is possible that the greater reduction of false memories with tDCS was due to the disruption of gist formation during the encoding phase, in addition to being more literal in the retrieval phase.

One important limitation of our study is that we did not evaluate the effects of tDCS of other cortical areas on false memories. Therefore we cannot confirm that the effects observed here are specific to the stimulation of the left temporal cortex with anodal tDCS. Besides this limitation, it is valuable to discuss the potential application of this technique. The rTMS study of false memory [6] suggested a forensic application, by reducing false memories after an event has been encoded (such as in eyewitness testimony). In the present study the “event” to be remembered (list of words) was presented during stimulation. Our present study suggests that using tDCS to modify the encoding and retrieval of memories is a good candidate for facilitating the acquisition of new information by reducing false memories.
